# Supplementary material for: The network signature of constellation line figures
Source: PLoS One. 2022 Jul 28;17(7):e0272270. doi: 10.1371/journal.pone.0272270 (PMC9333327; doi:10.1371/journal.pone.0272270)
Supplement: Attachment — Submitted filename: Rebuttal letter.pdf [file pone.0272270.s001.pdf]

# Rebuttal letter PONE-D-21-36117
# The network signature of constellation line figures

In this letter, the reviewers' comments are pasted in green, and my answers in black. The letter starts with brief answers to the reviewers' comments, and ends with a longer summary of the improvements made to the manuscript, which go beyond the reviews to an extent. In the marked-up copy of the revision, the paragraphs with significant changes are marked with yellow lines.

---

## Reviewers' comments

Reviewer #1: The authors performed a massive study of star constellations in an attempt to correlate line figures with distinct cultures across human history. They explained their methodology - based on network science - and their results in great detail. Because they were very ambitious in their goals, which is good, the authors failed to **provide a clear message** to the readers. Unless the reader can make notes and pay great attention to the details and figures, he/she cannot infer which the main conclusions are. All one can learn is that constellation line figures can be classified according various criteria and the classification correlates poorly with cultures. Some distinction can be made between oral and written astronomies.

Therefore, a recommend a major revision for the manuscript to be useful in conveying a message to PLOS One readers, as follows.
1) The **Abstract** should make it explicit how network science is applied to get the results. This piece of information is hidden.
2) The authors should select **a few items for comparison** in which there is either a high or no correlation, so that the conclusions could be more easily understandable by the reader.
3) The **Conclusion** section ends abruptly with description of methodology, and the reader is left without a comment about the implications of the results.

1) The **Abstract** was rewritten to include the crucial technical keywords: embedding, clustering, network of constellations, assortativity over this, diversity/entropy. Before, the method was not described in the abstract. Now, it has a summary there.
2) Both the **Introduction** and the **Discussion and conclusion** sections were rewritten, such that they include the key results as a takeaway message.
3) In particular, the **Discussion and conclusion** section was rewritten such that it contains 3 paragraphs titled "Implications of the results". There, the implications are made clearer, in sentences "These findings imply…", for example *"We found relatively universal line designs in a minority of sky regions around popular stars in IAU Cas, CrB, and Sco. Long chains of stars are drawn in these sky regions regardless of what the constellation represents: these shapes appear also when the constellation does not represent the scorpion which recurs across cultures in the IAU Sco region. This implies that geometry is even more universal than semantics in these sky regions".*

Perhaps more importantly, the manuscript was reorganised, such that the Results start very quickly (on line 110, or page 4 in the manuscript version including the inline figures). The results are also expanded, and are described with less obfuscation than before.

Reviewer #2: This is one of the most exciting papers I have read in the last 5 years. I would like to thank the author for the novel approach and study of an interesting topic. I personally learned a lot and would like to see this work published with some improvements.

Thank you for writing this. It provided further motivation to improve the manuscript.

I have also learnt a lot from doing this work (and made new friends in other sciences: astronomy and cognitive sciences). Also, the quantitative results it provided (for example, the diversity numbers in Hypothesis II) are fairly surprising, given the existing literature which chose to emphasise the cultural similarities (on the lines of: the Big Dipper and Orion's belt are pervasive asterisms), but not the differences.

I find the methodology solid in terms of feature extraction and projection into low dimensional space for visual analysis. My only concern is that almost all of the hypotheses are supported by the interpretation of the tSNE projections.

It is likely a fault of how I wrote the original submission, but: it is not the case that the hypotheses are supported by the tSNE projection. There is the "original" dataset in 19 dimensions (features of constellations). It is on this dataset that all numerical results for Hypothesis I are obtained. In other words, all measurements in **I.1-4** are done before projection to two dimensions. I had made a note of that in the text, but it was easy to miss it.

I have now clarified the matter in the manuscript. The method is essentially this: for Hypothesis I, the projection is useful to deliver the intuition to the reader. This intuition is not achievable otherwise, using the data in 19 dimensions. The measurements (assortativity r, similarity Δ) are instead done in the 19 dimensions, because it is perfectly feasible to do so. The results are only explained using two-dimensional visualisations. This is sound, because the projection is of good quality (trustworthiness 0.98 out of 1), meaning that it preserves neighbourhoods well, so can act as an explainer. I could also have presented the same numerical results completely without the projection and its visualisations. It would simply have been extremely… abstract.

Now, it was also perfectly possible to run the analysis also on the projected dataset in 2 dimensions. But, this essentially introduces small errors due to the embedding algorithm. I have done this, too, and the results are similar (within some 2% of the results obtained over 19 dimensions).

I think the author can improve the approach by creating ML models and use measures of performance to support relevant research questions. For instance (1) classification problem [...]

An ML-based method was my initial thought, but it only suited some of the questions, where the number of classes is reasonably small. In Hypothesis I, the number of classes is first 56, then 4, then 2, then 9. The first is too high to train a classifier on, using only the <2000 data points (constellations) available to both train and test.

This is why I settled on a method based on fundamental statistics (like the assortativity coefficient) which works regardless of the number of categories in the data.

Nevertheless, I did find a good use for a **classifier** in the revision: for Question I.2. Astronomical literacy, I had claimed that a particular feature (magnitude) distinguishes oral from written constellations – but I hadn't proven it.

So, for that question, I now added a classifier (SVC with a nonlinear kernel, but any nonlinear classifier I tried worked just as well). This classifier is trained to distinguish oral from written, and provides (1) the balanced accuracy (0.75, quite good), and (2) the most important feature to distinguishing oral from written (which is indeed average magnitude). I also report per-class F1 scores, because they help explain which class is easier to predict (written!).

So that was useful, and it is now added in the Results section, although on only a few lines.

…or (2) applying clustering to the data and measuring accuracy using the labeled data (e.g., in Fig5).

It may look like the clusters in Fig 5 (now Fig 4 in the revision) are delineated by hand, but it's not the case: they really correspond to the clusters (technically *strongly connected components*, meaning groups of constellations that are disconnected from each other) automatically extracted from the network of constellations. I added a note to that end in the revision.

In my opinion, the paper could also benefit from restructuring, as the background information and interpretations are very text-heavy and make it harder to follow. Perhaps the authors could **focus on the most striking results first** and then provide descriptive statistics of the analysis.

This was useful. To solve the problem, I restructured the paper. Instead of the previous section order

Data -> Method -> Results -> In-depth data and method

I now have simply:

Results -> Data -> Method.

(This is the order required by Nature journals, for example. All results come first.)

The text was appropriately moved to support this order. The Results start right after the Introduction, and they can be read independently of the Data and Method sections. The text in Results is also friendlier to the reader, as a consequence.

Also, the Introduction section has 2 paragraphs with summaries of the most important results.

Further improvements were made to the manuscript, as listed below.

---

## Summary of improvements

**Major changes:**

- ➔ In the time until revision, the data increased by 12%: from 1591 to **1802 constellations** with lines, and from 50 to **56 cultures**.

  Of these new cultures, 4 are Pacific islands of Polynesian or neighbouring ancestry (**Caroline**, **Kiribati**, **Manus**, and **Marshall** Islands) so that region is now much better represented. This data was hiding in unusual places, such as two language dictionaries. Another new culture is the **Huave** indigenous (but contemporary) culture of Mexico. The final is the Charles **Dien** star chart (Paris Observatory, 1831), which marks the point when the line representations for constellations became popular in Europe (the same astronomer produced the charts in the popular French Flammarion atlases throughout the 19th century). In addition, a few variants of constellations were added to existing cultures, and minor corrections were applied (two lines were inaccurate).

  The manuscript was updated accordingly (all figures have the new cultures and data points). The numerical results remain within 2% of the previous values. There is a new insight: the **Marshall** Islands constellations resemble the Chinese ones, so there is now a better similarity link Polynesia-China. Also this same culture "fills" some space in the embedding which was previously without data points, and thus the smallest cluster of constellations (was called C3) has now been united into its large neighbour, C2. The map of constellations was updated.

  *(Note: There will be a separate publication documenting the dataset formally, with: an accessible data format, scholarly sources provided per sky object, marks for the certainty / quality of each data object, various metadata per constellation and per culture, including semantics / meaning – features that this manuscript does not study. With this information, the data can be reused by other scientists, across disciplines. That documentation is a very complex, headache-inducing problem, and far too long to include in this manuscript. This dataset will be a living dataset, open to contributions. It is hoped that even more data survives in some form, and can be digitised.)*

- ➔ The manuscript was **restructured**. The sections appear in the order **Intro -> Results -> Data -> Method**. Since the methodological details come last, the previous Fig. 3 (a small summary of the method) was removed.

- ➔ A **classifier** was added as a supplementary method to dig into the details, for Question I.2 (see Results section, from line 266).

- ➔ Many paragraphs were rewritten, including in particular the Abstract, Introduction, and Discussion and conclusion. More paragraphs were added to the discussion of results also in the Results section.

**Minor changes:**

➔ The names of the cultures were homogenised to be **nouns** rather than adjectives, as much as possible: place names (Anuta not Anutan, China not Chinese), author names (Rey, IAU, Al-Sufi), and population or tribe names (Blackfoot, Huave). Some were originally adjectives for historical reasons (older data from the astronomical package Stellarium had such names). The nouns are much more useful when a reader wants to locate cultures geographically, particularly small places which are not commonly known. The only exception remains the adjective "Western", for which there's no place name.

➔ Wrote the statistical error $\sigma_r$ using powers of zero (for example $\sigma_r < 10^{-3}$), so the text is neater in the Results section.

➔ Transformed some absolute numbers ("1412 constellations") into relative ones ("78% of the total"), which should be more memorable to the reader.

➔ The text was edited down as much as possible, particularly the in-depth background text. Despite all the additions around the new data, the manuscript is now shorter than the first draft (minus 15 lines of text, minus one small figure).

➔ Typo corrected: 219 instead of 210 Korean constellations in Table 1. This was a mistake.

➔ The link to the data has changed, to a more permanent and findable GitHub repository [https://github.com/doinab/constellation-lines].

➔ "Fig." is now "Fig" (no dot), as per journal guidelines.

---

*With many thanks for your help in polishing this text,*

D. Bucur (University of Twente, The Netherlands)
